# Efficacy of silver needle therapy for the treatment of chronic nonspecific low back pain: a prospective, single-center, randomized, parallel-controlled clinical trial

**DOI:** 10.1186/s13063-021-05040-y

**Published:** 2021-01-21

**Authors:** Xuesong Hu, Shaoxing Dong, Bing Zhang, Xuan Wang, Yanwei Yin, Chuansheng Liu, Junmin Yu, Xing Wu, Fenghu Xu, Chao Meng

**Affiliations:** 1 Department of Pain, the People’s Hospital of Yuxi City, The 6th Affiliated Hospital of Kunming Medical University, Yuxi, China; 2 Department of Anesthesiology, The Affiliated Hospital of Qingdao University, Qingdao, China; 3 Department of Pain Management, The Affiliated Hospital of Qingdao University, No. 1677, Wutai Mountain Road, Huangdao District, Qingdao City, 266000 Shan Dong Province China

**Keywords:** Silver needle therapy, Physiotherapy, Chronic nonspecific low back pain

## Abstract

**Background:**

Chronic nonspecific low back pain (CNSLBP) troubles approximately 30% of people worldwide. Silver needle therapy (SNT) is a treatment method to relieve soft tissue pain through heating. Therefore, this study aimed to observe the effects of SNT on CNSLBP.

**Methods:**

In this study, 100 patients were randomly divided into 2 groups: silver needle (SN) group and control group (*n* = 50). In the SN group, patients received SNT and physiotherapy, while patients received physiotherapy alone in the control group. At the 6-month follow-up, the numerical rating scale (NRS), Oswestry Disability Index (ODI), Short-Form 12 of quality of life (SF-12), the natural logarithms of low-frequency measurement (InLF), and the natural logarithms of high-frequency measurement (InHF) of heart rate variability (HRV) were recorded.

**Results:**

In both groups, NRS, ODI, SF-12 scores, and HRV at 2 weeks after treatment were improved and maintained for 6 months. Compared with the control group, more significant improvements were observed in the NRS and SF-12 scores at 1, 2, 3, and 6 months and in the ODI scores at 1 and 2 months in the SN group (*P* <  0.05). However, there was no significant difference between the groups in the ODI scores at 3 and 6 months. InLF and InHF in the SN group were higher than those in the control group at 3 and 6 months (*P* <  0.05).

**Conclusions:**

SNT relieved pain and improved quality of life and autonomic nerve activity, especially parasympathetic nerve, in patients with CNSLBP, without serious complications.

**Trial registration:**

Chinese Clinical Trial Registry No. ChiCTR-OOC-17013237. Registered on November 11, 2017.

## Introduction

Low back pain induced by fasciitis and facets mostly is a worldwide growing problem affecting the quality of life of 30% of the population, with 85% cases categorized as nonspecific low back pain [1]. Among these cases, 8% develop into chronic nonspecific low back pain (CNSLBP), defined as low back pain that lasts more than 3 months [2, 3] and that may lead to life inconvenience and psychological problems, such as pain-related disability, poor sleep, depression, and anxiety [4]. Therefore, treatment of CNSLBP is important and necessary.

Currently, physical therapy [5], pulsed electromagnetic field therapy [6], opioid therapy [7], and nonsteroidal anti-inflammatory drugs (NSAIDs) [8] are used for CNSLBP. However, these methods still could not achieve good effects. Acupuncture, as a traditional Chinese treatment, has shown a significant therapeutic effect for many types of diseases, including CNSLBP [9]. Silver needle therapy is similar to traditional acupuncture but has some differences. Silver needle is placed in muscles, tendons, and fascia instead of at acupoints, and the needle is heated using a special machine to eliminate aseptic inflammation and to relieve pain [10]. Silver needle therapy decreases pain mainly through 3 mechanisms: elimination of sterile inflammation, improvement of blood circulation, and relief of muscle spasms [11]. The mechanisms of pain control that underlie silver needle therapy are similar to those of moxibustion, but moxibustion is heated by burning cotton balls, which cannot control the temperature. The silver needles were heated by special equipment that set the temperature according to the patients’ feedback. Therefore, the primary objective of this trial was to assess the effects of silver needle therapy on CNSLBP.

## Patients and methods

### Patients

This study was registered in the Chinese Clinical Trial Registry, and the registration number is ChiCTR-OOC-17013237. This study was approved by the Medical Ethics Committee, and all patients provided their informed consent.

**Fig. 1:**
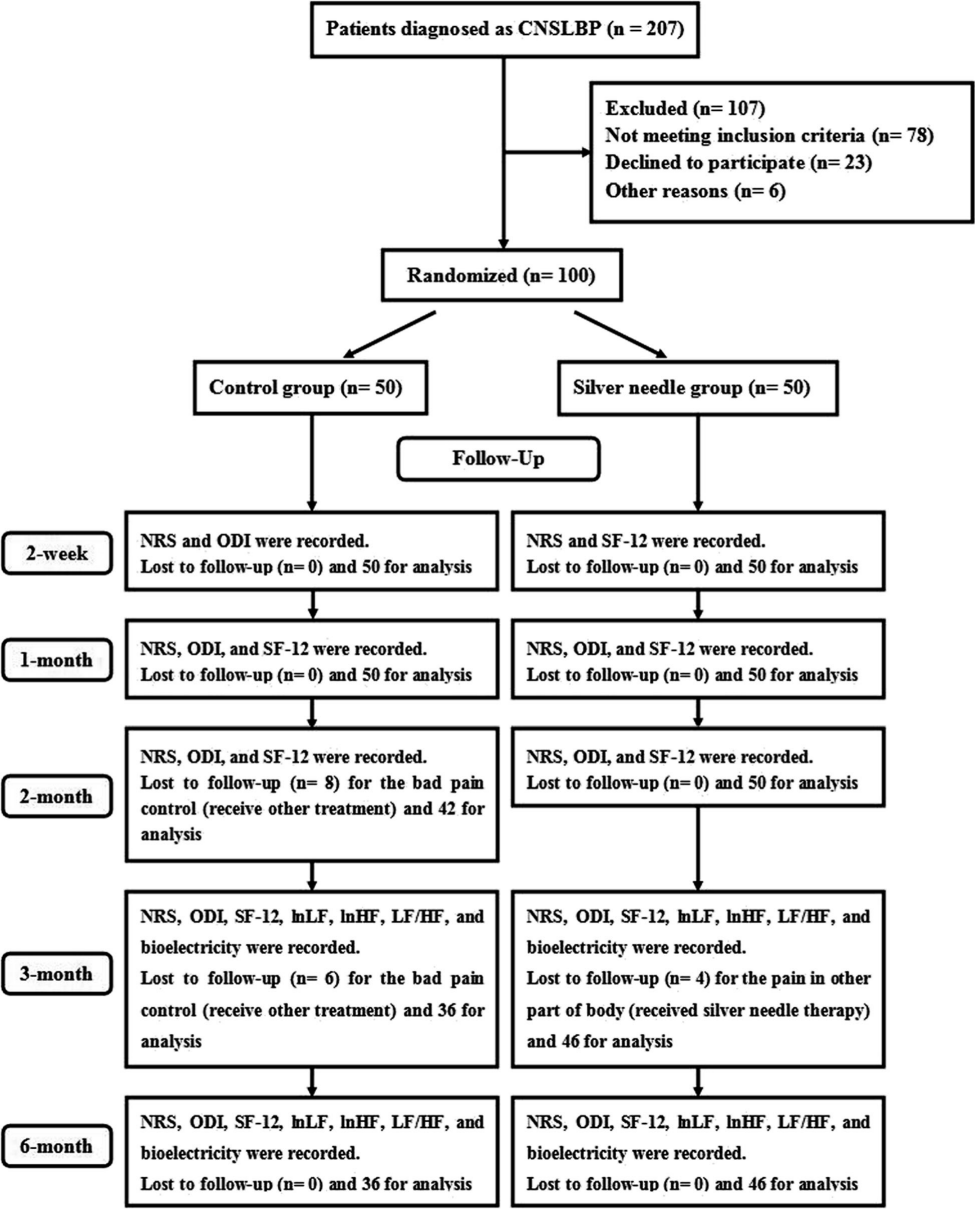
Flow diagram

From November 2017 to April 2018, patients who were diagnosed with CNSLBP [12] in the Department of Pain Management of the Affiliated Hospital of Qingdao University were recruited in this study and divided into 2 groups using the Stratified Blocked Randomization method: silver needle group (SN group) and control group (C group). The patients were recruited through advertisements or physicians. Inclusion criteria include the following: (1) CNSLBP (Diagnosis: the discomfort from the second lumbar vertebra to the bilateral sacroiliac joints and adjacent tissues, centered on the lumbosacral joint, which lasts more than 3 months; no systemic diseases such as tumors and tuberculosis, no mental illness, and no neurological involvement requires surgery); (2) age from 18 to 80 years old; (3) American Society of Anesthesiologists (ASA) grade I to III; and (4) provision of signed informed consent. Exclusion criteria include the following: (1) communication disorders, (2) coagulation abnormality, (3) local or systemic infection, (4) cardiovascular disease, (5) allergy to anesthetics, (6) secondary pain including cancer and ankylosing spondylitis; and (7) pregnancy (Fig. 1).

#### SN group

**Fig. 2:**
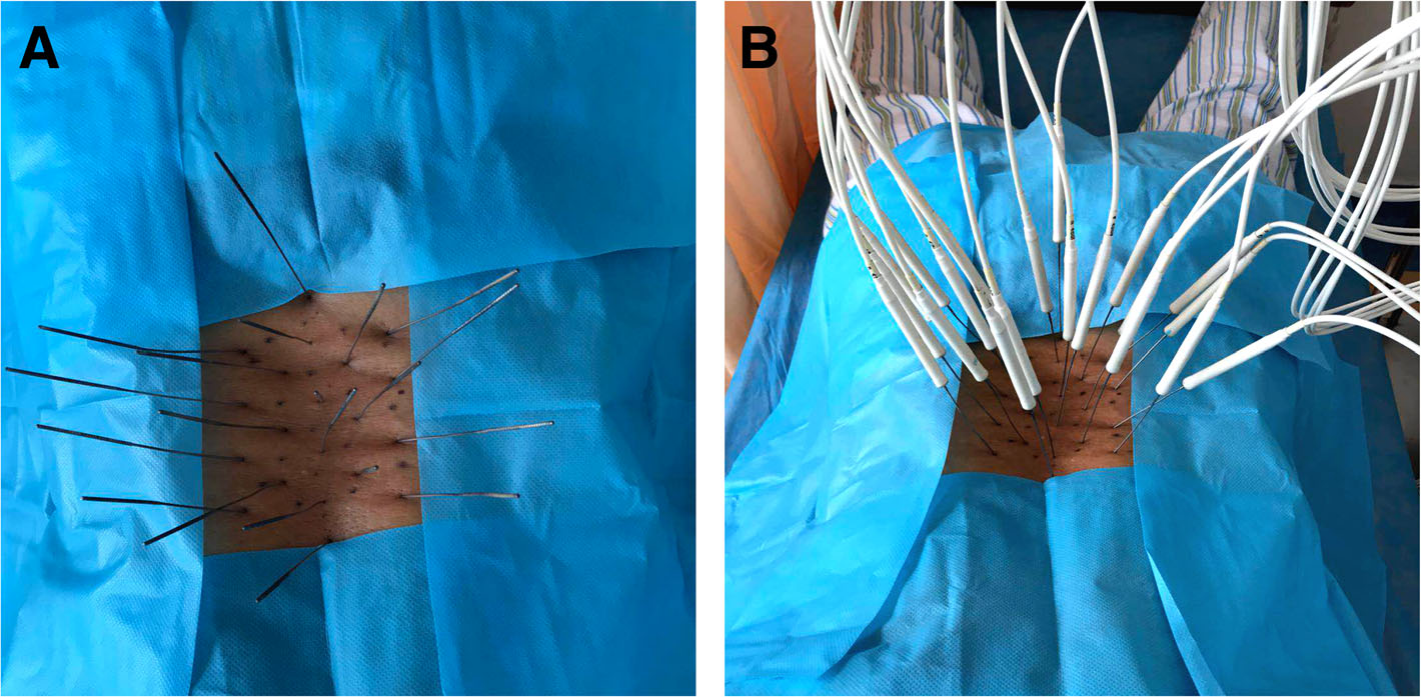
Silver needle therapy. **a** The silver needles were punctured into the interspinous ligament and paravertebral space in the area of the pain and/or the third lumbar transverse process for some patients with tenderness of the third lumbar transverse process. **b** The silver needles were connected to a special machine and heated for 25 min

After the relative checks and tests completed (including but not limit to the lumbar MRI, hematology, and hemagglutination), patients in this group received NSAIDs, medications like sodium aescinate that promotes blood circulation, and physiotherapy. Silver needle therapy (Fig. 2) was also performed, and the procedure was as follows:

1. The patient was placed in a prone position, and the precise pain points were determined by pressing with the thumb and marking the locations, which may be located at the point of muscle attachment, in the muscle bundle, fascia, or tendon.
2. With the precise pain points located at the center, each needle point was marked 1.5 cm apart around the center, eventually covering the pain area.
3. After sterilization, 0.5% lidocaine was injected intradermally according to the marked points. Then, the silver needles (17 cm length of needle body and 1 mm of diameter) were inserted perpendicularly or obliquely to the targeted points, which were usually by the attachment of the muscle or of the fascia to the bone surface. Most patients might inform the physician of soreness or a feeling of distention, indicating the correct positioning of the silver needle.
4. Then, the silver needles (SHANXI ASTRONANTICS IN GOAL DIRECTION MED TECH Co., LTD., Shanxi, China) were connected to a special machine (YW-L1000; SHANXI ASTRONANTICS IN GOAL DIRECTION MED TECH Co., LTD., Shanxi, China), and the heating temperature was set to ensure that the temperature of the silver needle entering the skin was approximately 42 °C for 25 min.
5. The silver needles were withdrawn after they had cooled. Sterilization was performed again. The patients were instructed to keep the needled skin away from water for at least 3 days.

#### C group

After the relative checks and tests were completed, the patients in this group received NSAIDs and medications that promote blood circulation, similar to the SN group. The patients also received physiotherapy, including but not limited to polarized light and ultrasonic drug penetration therapy.

### Measurements

The demographic data of all patients were recorded. The primary outcome was the 10-point numerical rating scale (NRS) at 2 weeks and 1, 2, 3, and 6 months after the treatment. The secondary outcomes were Oswestry Disability Index (ODI) at 2 weeks and 1, 2, 3, and 6 months after the treatment, the Short-Form 12 of quality of life (SF-12) at 1, 2, 3, and 6 months after the treatment. The bioelectricity (Lx-03-01, Zhi Kang Technology Co., Ltd., Beijing, China) and heart rate variability (HRV) (ZSY-1 Heart Rate Variation Detector, Wegene Technology Inc., Shenyang, China) [13], including the natural logarithm of the low-frequency measurement (InLF), the natural logarithm of the high-frequency measurement (InHF), and the low frequency/high frequency ratio (LF/HF) were also assessed before the treatment and 3 and 6 months after the treatment. Additionally, complications, including aggravation of pain, local hematoma, infection, and local anesthetic toxicity, were also recorded. The physicians who collected and analyzed data were blinded.

### Statistical analysis

According to the NRS at 2 weeks after treatment (3.5 ± 1.29 in the SN group, 4.25 ± 0.96 in the C group, *n* = 4) in the pilot trial, a sample size of 36 patients per group was required to provide 80% power to detect differences at an *α* level of 0.05, indicating significance. However, to prevent a 35% attrition rate, we will eventually recruited 100 patients with 50 in each group.

The quantitative data, including age, body mass index (BMI), NRS, ODI, SF-12, bioelectricity score, and InLF, InHF, and LF/HF of HRV were presented as the means ± standard deviation and compared using a *t*-test. The count data, including sex, ASA grade, complications, and analgesic intake, were presented as frequencies and percentages and were compared using the chi-squared test, Fisher’s exact test, or R*C chi-square test. The repeated data were analyzed by repeated-measures analysis of variance. *P* <  0.05 was considered statistically significant. SPSS 20.0 was used to perform statistical analysis.

## Results

### Patients for analysis

There were 207 patients diagnosed with CNSLBP during the trial, while 107 did not meet the inclusion criteria. In total, 100 patients were included in this trial and were randomly divided into 2 groups (*n* = 50). At 2 weeks and 1 month, all patients in each group were included for analysis. However, 8 patients in the C group requested other active treatments due to bad pain control, and thus 42 patients in the C group were included for analysis at 2 months. Fifty patients were included in the SN group. At 3 months, another 6 patients in the C group with bad pain control requested other treatments, and 4 patients in the SN group with pain in other parts of the body requested silver needle therapy so that 36 patients in the C group and 46 in the SN group were included in the analysis. The same number of patients was included at 6 months (Fig. 1).

### Demographic data

The age of patients in the SN group (50.8 ± 15.3 years old) showed no significant difference compared with that in the C group (48.9 ± 14.3 years old). The BMI, sex, ASA grade, NRS, ODI, SF-12, bioelectricity score, and InLF, lnHF, and LF/HF of HRV of the patients in the two groups also were not significantly different (Table 1).

**Table 1:** Demographic data (*n* = 50)

	Control group	Silver needle group
Female/male (number)	26/24	28/22
Age (year)	48.9 ± 14.3	50.8 ± 15.3
BMI (kg/m^2^)	24.3 ± 3.0	24.8 ± 2.4
ASA grade		
I	28	24
II	15	20
III	7	6
NRS score	8.02 ± 0.80	7.90 ± 0.91
ODI score	64.8 ± 2.8	65.6 ± 2.7
SF-12 score	19.7 ± 3.0	19.0 ± 3.3
Bioelectricity score	60.6 ± 4.4	60.4 ± 3.4
InLF (In (ms^2^))	4.03 ± 0.59	3.99 ± 0.66
InHF (In (ms^2^))	3.42 ± 0.56	3.39 ± 0.59
LF/HF	1.85 ± 0.27	1.84 ± 0.26

The demographic data of patients in the two groups had no significant difference

*BMI* body mass index, *ASA* American Society of Anesthesiologists, *NRS* numerical rating scale, *ODI* Oswestry Disability Index, *SF-12* Short-Form 12 of quality of life, *lnLF* natural logarithms of the low frequency measurement, *lnHF* natural logarithms of the high frequency measurement, *LF/HF* low frequency/high frequency ratio

### Silver needle therapy decreased the NRS scores of patients with CNSLBP

**Fig. 3:**
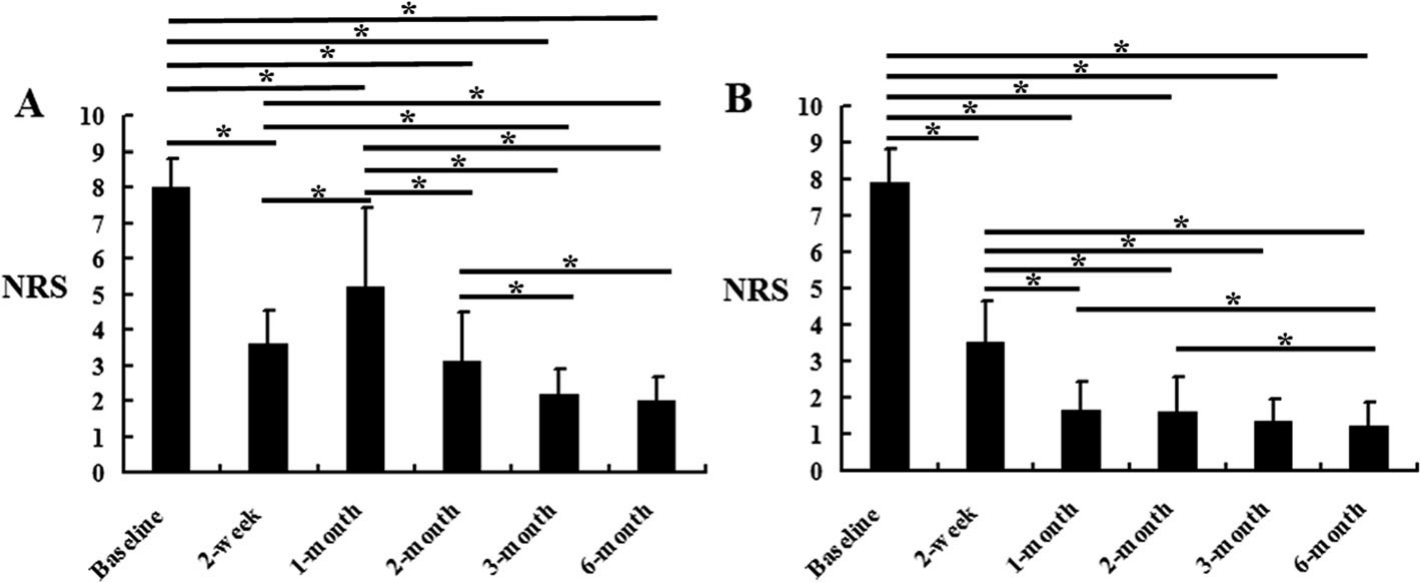
The NRS results in the control group and silver needle group. This figure shows the comparison of NRS scores within the same group according to the time. **a** The control group. **b** The silver needle group. ^*^P < 0.05

Within the C group, the NRS scores decreased significantly at 2 weeks (3.60 ± 0.93), 1 month (5.22 ± 2.22), 2 months (3.10 ± 1.41), 3 months (2.19 ± 0.71), and 6 months (2.00 ± 0.68) compared with the baseline scores (8.02 ± 0.80) (*P* <  0.05). However, compared with 2 weeks, the NRS scores increased significantly at 1 month (*P* <  0.05). At 2, 3, and 6 months, the NRS scores decreased compared with the 1 month scores, and the NRS scores at 3 and 6 months were lower than those at 2 months (*P* <  0.05). The NRS scores at 3 and 6 months showed no significant difference (Fig. 3a). Within the SN group, the NRS scores decreased significantly at 2 weeks, 1, 2, 3, and 6 months compared with the baseline scores (*P* <  0.05). Compared with 2 weeks, the NRS scores decreased at 1, 2, 3, and 6 months (*P* <  0.05). However, the NRS scores at 3 and 6 months showed no significant difference (Fig. 3b). Additionally, the NRS scores at 1, 2, 3, and 6 months in the SN group were lower than those in the C group (*P* <  0.05) (Table 2).

**Table 2:** Comparison of NRS and ODI scores in the control group and silver needle group (mean ± SD)

		Control group	Silver needle group	*P* value
NRS score (reduction rate)	Baseline	8.02 ± 0.80	7.90 ± 0.91	0.484
	2 weeks	3.60 ± 0.93 (54% ± 12%)	3.52 ± 1.13 (55% ± 15%)	0.699
	1 month	5.22 ± 2.22 (35% ± 28%)	1.64 ± 0.78 (79% ± 11%)	< 0.001
	2 months	3.10 ± 1.41 (61% ± 18%)	1.62 ± 0.95 (79% ± 12%)	< 0.001
	3 months	2.19 ± 0.71 (73% ± 10%)	1.33 ± 0.63 (83% ± 9%)	< 0.001
	6 months	2.00 ± 0.68 (75% ± 11%)	1.22 ± 0.66 (84% ± 9%)	< 0.001
ODI score	Baseline	64.7 ± 2.8	65.6 ± 2.7	0.147
	2 week	39.4 ± 3.2	39.8 ± 3.3	0.584
	1 month	44.5 ± 16.7	27.2 ± 3.4	< 0.001
	2 months	30.3 ± 8.6	24.3 ± 2.2	< 0.001
	3 months	24.6 ± 2.1	24.6 ± 3.0	0.901
	6 months	25.1 ± 2.4	24.8 ± 3.0	0.709

*NRS* numerical rating scale, *ODI* Oswestry Disability Index, *Reduction rate* compared with the baseline, the reduction degree of NRS

### Silver needle therapy improved the ODI and SF-12 scores for patients with CNSLBP

**Fig. 4:**
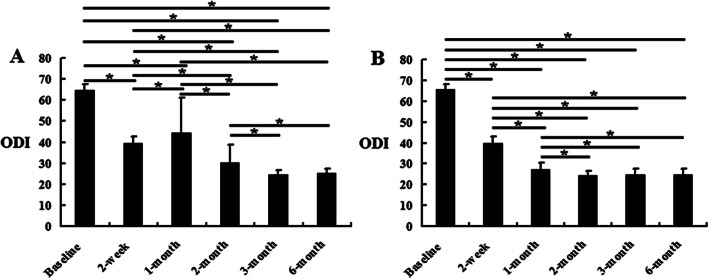
The ODI results in the control group and silver needle group. This figure shows the comparison of ODI scores within the same group according to the time. **a** The control group. **b** The silver needle group. ^*^P < 0.05

**Fig. 5:**
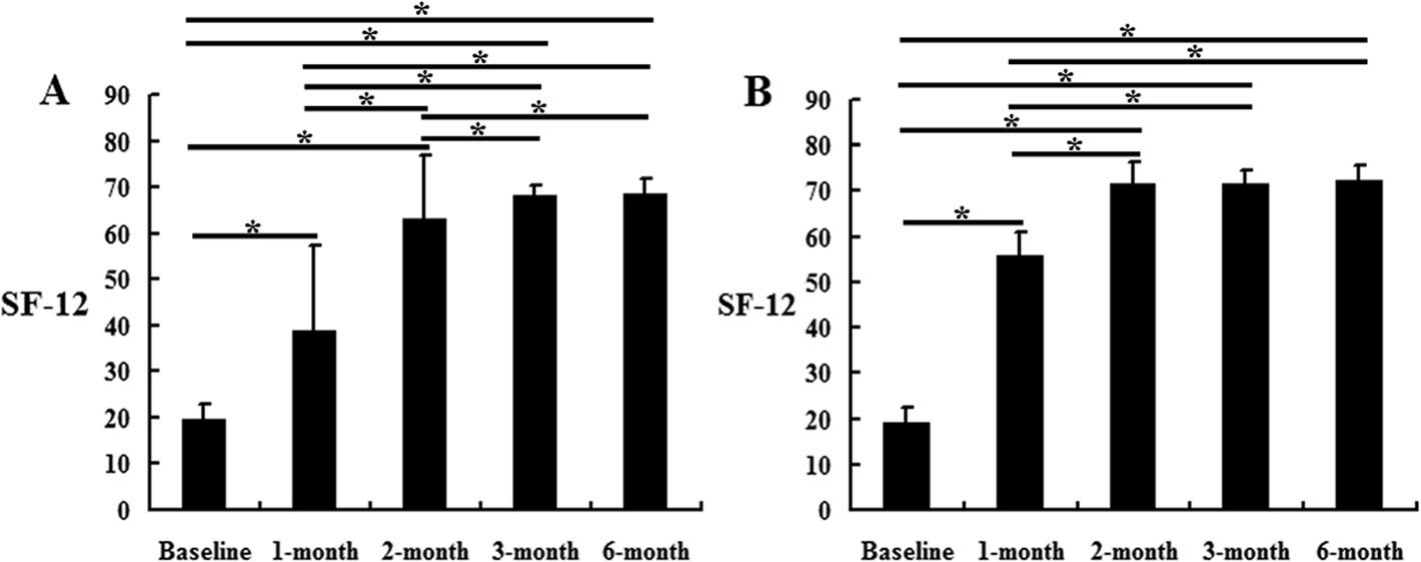
The SF-12 results in the control group and silver needle group. This figure shows the comparison of SF-12 scores within the same group according to the time. **a** The control group. **b** The silver needle group. ^*^P < 0.05

Within the C group, the ODI scores at 2 weeks, 1, 2, 3, and 6 months were lower than the baseline scores (*P* <  0.05). Compared with 2 weeks, the ODI scores at 1 month increased significantly (*P* <  0.05). Compared with the 1 month scores, the ODI scores at 2, 3, and 6 months decreased significantly, and the ODI scores at 3 and 6 months were lower than those at 2 months (*P* <  0.05). However, the scores at 3 and 6 months were not significantly different (Fig. 4a). Within the SN group, the ODI scores decreased significantly at 2 weeks and 1, 2, 3, and 6 months compared with the baseline scores, and the ODI scores at 1, 2, 3, and 6 months were lower than those at 2 weeks (*P* <  0.05). Compared with the 1 month scores, the ODI scores further decreased at 2, 3, and 6 months (*P* <  0.05). However, the ODI scores at 2, 3, and 6 months showed no significant difference (Fig. 4b). Additionally, the ODI scores at 1 and 2 months in the SN group were lower than those in the C group (*P* <  0.05), while at 3 and 6 months, there was no difference (Table 2). The SF-12 results showed an opposite trend to that of the ODI scores in the C and SN groups. However, the SF-12 results at 3 and 6 months in the SN group were higher than those in the C group (Table 3 and Fig. 5) (*P* <  0.05).

**Table 3:** Comparison of SF-12 scores in the control group and silver needle group (mean ± SD)

	Control group	Silver needle group	*P* value
Baseline	19.7 ± 3.0	19.0 ± 3.3	0.251
1 month	38.7 ± 18.6	55.7 ± 5.2	< 0.001
2 months	63.0 ± 13.9	71.8 ± 4.5	< 0.001
3 months	68.1 ± 2.4	71.7 ± 2.7	< 0.001
6 months	68.7 ± 3.0	72.4 ± 3.2	< 0.001

*SF-12* Short-Form 12 of quality of life

### Silver needle therapy improved the bioelectricity and HRV scores

**Fig. 6:**
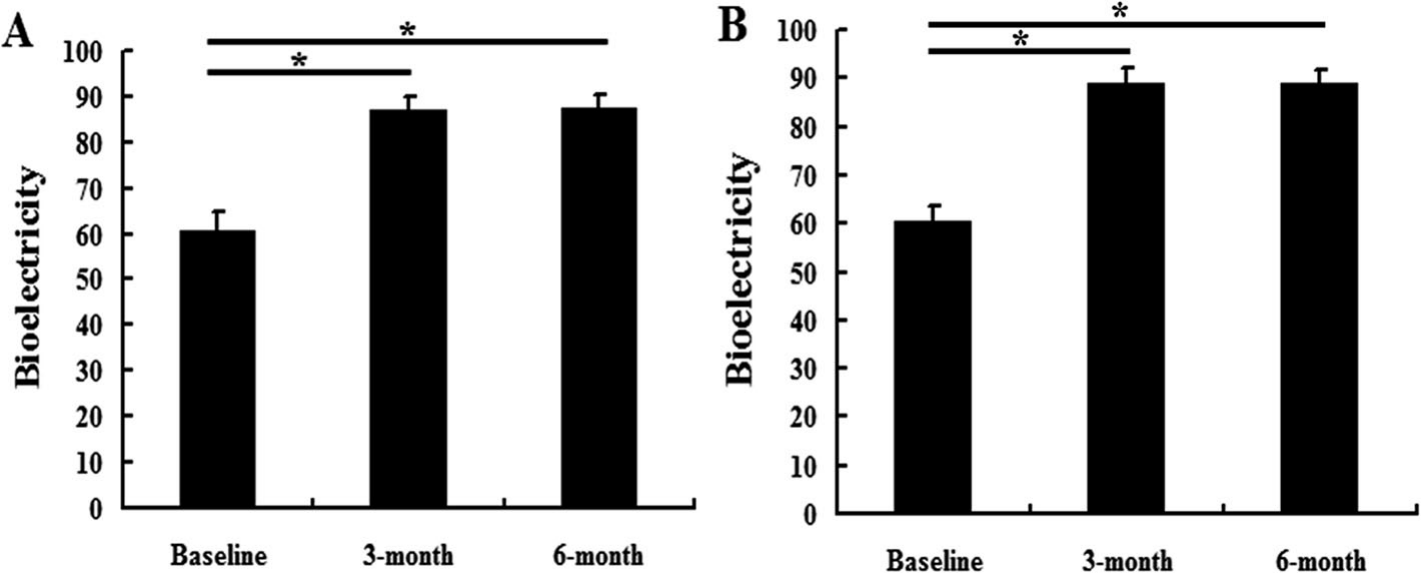
The bioelectricity results in the control group and silver needle group. This figure shows the comparison of bioelectricity scores within the same group according to the time. **a** The control group. **b** The silver needle group. ^*^P < 0.05

**Fig. 7:**
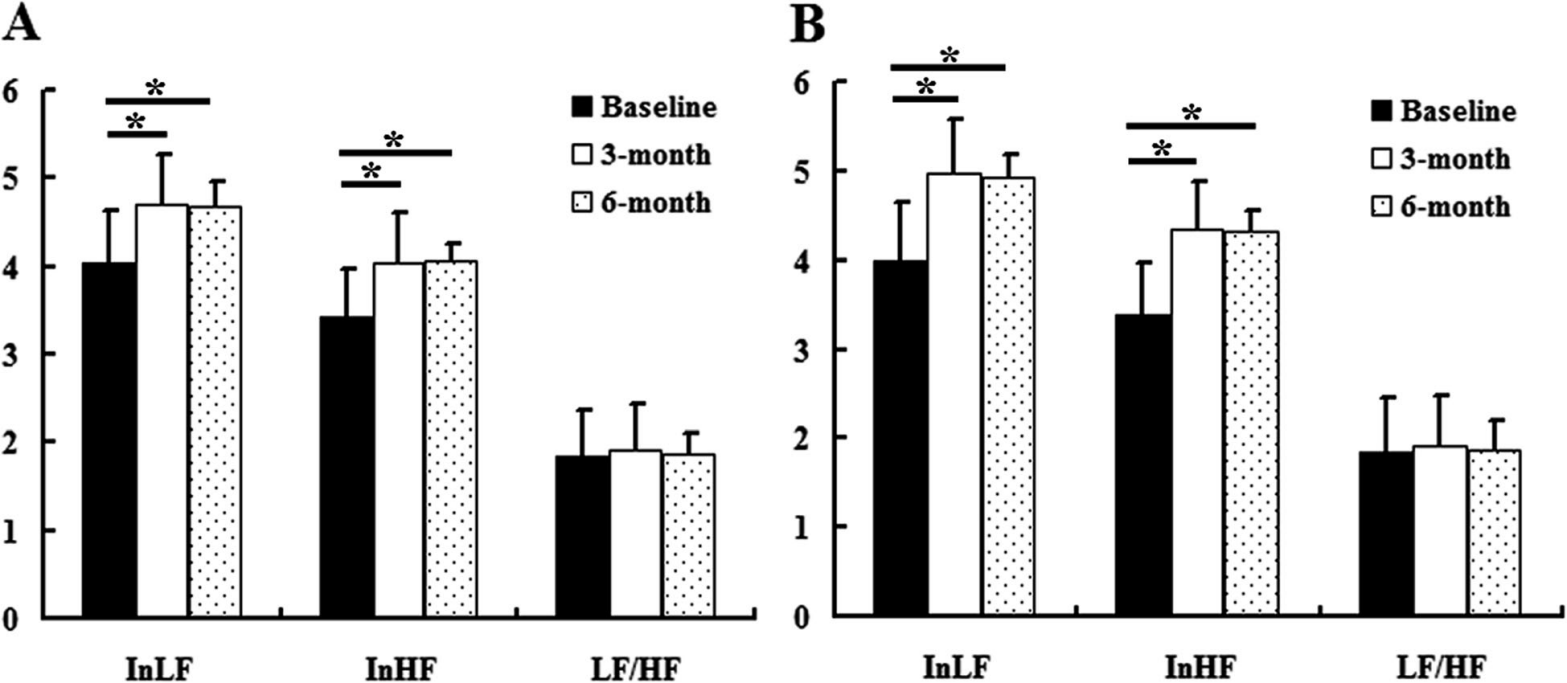
The HRV results in the control group and silver needle group. This figure shows the comparison of InLF, InHF, and LF/HF of HRV within the same group according to the time. **a** The control group. **b** The silver needle group. ^*^P < 0.05

Within the C and SN groups, the bioelectricity scores at 3 and 6 months were higher than the baseline scores (*P* <  0.05), but the scores at 3 and 6 months were not significantly different (Fig. 6). Compared with the C group, the bioelectricity scores in the SN group were higher at 3 and 6 months (*P* <  0.05) (Table 3). The InLF and InHF displayed a similar result as the bioelectricity scores (*P* <  0.05), while the LF/HF did not differ between the 2 groups at 3 and 6 months (Table 4 and Fig. 7).

**Table 4:** Comparison of bioelectricity and HRV scores in the control group and silver needle group (mean ± SD)

		Control group	Silver needle group	*P* value
Bioelectricity score	Baseline	60.6 ± 4.4	60.4 ± 3.4	0.763
	3 months	86.9 ± 3.0	88.8 ± 3.3	0.007
	6 months	87.3 ± 3.2	89.1 ± 2.7	0.008
InLF (In (ms^2^))	Baseline	4.03 ± 0.59	3.99 ± 0.66	0.738
	3 months	4.70 ± 0.54	4.98 ± 0.58	0.025
	6 months	4.68 ± 0.51	4.93 ± 0.62	0.047
InHF (In (ms^2^))	Baseline	3.42 ± 0.56	3.39 ± 0.59	0.778
	3 months	4.05 ± 0.55	4.34 ± 0.54	0.019
	6 months	4.06 ± 0.51	4.32 ± 0.55	0.028
LF/HF	Baseline	1.85 ± 0.27	1.84 ± 0.26	0.748
	3 months	1.92 ± 0.20	1.92 ± 0.24	0.952
	6 months	1.87 ± 0.23	1.87 ± 0.32	0.988

*HRV* heart rate variability, *lnLF* natural logarithms of the low frequency measurement, *lnHF* natural logarithms of the high frequency measurement, *LF/HF* low frequency/high frequency ratio

### Complications and medication intake

At 1 month after treatment, there were no complications in the C group, but there were 5 patients with complications in the SN group: 4 with the ecchymosis, and 1 with ambustion. However, there was no significant difference in the rate of complications between the two groups. We also recorded the number of patients who took medications at 1 month, and the number of patients in the SN group (4) was lower than that in the C group (25) (*P* <  0.05) (Table 5).

**Table 5:** Comparison of complication and medications intake in the control group and silver needle group

	Control group	Silver needle group	*P* value
Complications (Y/N)	0/50	5/45	0.066
Medications intake (Y/N)	25/25	4/46	< 0.001

### The results in SNT group analyzed by intention-to-treat (ITT)

The ITT analysis included all the patients, including those lost during the follow-up. The data of lost cases were carried forward and were set as the follow-up outcomes directly. That meant the previous follow-up results of the lost cases were regarded as the subsequent result. In this study, we used the comparison between the data including lost cases and the data excluding lost cases to perform ITT analysis and to determine whether the lost cases would affect the results. According to the flow diagram, the lost cases occurred at 3 months and 6 months in the SNT group, and we provided the outcomes of NRS, ODI, and SF-12 at 3 months and 6 months by ITT analysis. Due to the first detection of HRV occurring at 3 months and 6 months and because some cases have been lost, so the ITT analysis was not applied to the related outcomes of HRV. Compared with the SNT group excluding lost cases, the NRS, ODI, and SF-12 SNT group including lost cases had no significant difference (Table 6).

**Table 6:** Comparison between the data including lost cases and the data excluding lost cases in the SNT group

		SNT group including lost cases (***n*** = 50)	SNT group excluding lost cases (***n*** = 46)	***P***
**3 months**	**NRS**	**1.36 ± 0.66**	**1.33 ± 0.63**	**0.799**
	**ODI**	**24.6 ± 2.9**	**24.6 ± 3.0**	**0.921**
	**SF-12**	**71.7 ± 2.6**	**71.7 ± 2.7**	**0.980**
**6 months**	**NRS**	**1.26 ± 0.69**	**1.22 ± 0.66**	**0.760**
	**ODI**	**24.7 ± 2.9**	**24.8 ± 3.0**	**0.902**
	**SF-12**	**72.2 ± 3.1**	**72.3 ± 3.2**	**0.926**

## Discussion

In this study, silver needle therapy was associated with lower NRS scores and higher SF-12 scores than physiotherapy alone at 1, 2, 3, and 6 months for patients with CNSLBP. Silver needle therapy also increased the bioelectricity scores, InLF, and InHF at 3 and 6 months. However, compared with physiotherapy, silver needle therapy only decreased the ODI scores at 1 and 2 months without a significant difference at 3 and 6 months. In addition, the results of ITT analysis indicated that the results that got through ITT analysis and per-protocol analysis were similar, which meant that the lost cases did not affect the results of SNT.

In this study, silver needle therapy decreased NRS scores at 2 weeks and further decreased them at 1 month and maintained the decrease for 6 months in patients with CNSLBP. The ODI, a function index, achieved optimization at 2 months after silver needle therapy but showed no significant difference compared with physiotherapy at 3 and 6 months. Yan et al [14] found that moxibustion decreased pain in patients with discogenic low back pain. Song et al. [15] reported that moxibustion combined with other traditional medicines decreased the ODI scores of patients with headache, cervical pain, or lumbar pain. We observed that silver needle therapy decreased pain early and improved function later. This is because, after silver needle therapy, the muscle tissues recuperate within 1–2 months. Therefore, the optimal therapeutic effect for silver needle therapy was evident at 1–2 months after treatment. We also found that although silver needle therapy decreased NRS scores further at 3 and 6 months compared with physiotherapy, the ODI scores were not affected. Therefore, physiotherapy can also reduce pain to a level that patients can accept without affecting function.

In addition to the NRS and ODI scores, the SF-12, HRV, and bioelectricity scores were also detected. The SF-12 assesses quality of life in the previous month in many ways that include psychological and emotional factors [16], so we did not assess the SF-12 at 2 weeks after treatment. HRV is an index that accurately measures the autonomic nervous system (ANS) [13]. InLF represents ANS function, InHF represents parasympathetic nerve function, and LF/HF represents the sympathetic nerve function [13]. Bioelectricity is a comprehensive measurement through electrical bioimpedance detection technology to assess various physiological systems, including the patient’s nervous system. Detection of HRV and bioelectricity requires special instruments, and the patients needed to come to the hospital for the measurements. Therefore, we recorded these at 3 and 6 months to avoid losing more patients to follow-up.

Silver needle therapy was associated with a greater increased in SF-12 scores at 2 months and was maintained for 6 months after therapy. There have been many similar results. Fan et al [17] found that acupuncture improved the quality of life of patients with depression, assessed by the SF-36. Ren et al [18] found that moxibustion improved the physical and mental quality of life in patients with knee osteoarthritis also using the SF-36. The SF-12 and ODI both assessed functional outcomes, but the SF-12 scores were higher after silver needle therapy at 3 and 6 months than those following physiotherapy, while the ODI scores showed no significant difference. A possible reason for this finding was that some psychological factors involved in the SF-12 assessment could be affected by ANS. Silver needle therapy improved the function of ANS activity, mainly via the parasympathetic nervous system, as demonstrated by the increased InLF and InHF, and no significant changes in LF/HF were observed. We are well aware that ANS activity plays an important role in pain [19]. A previous study [20] showed that HF decreased (decreased parasympathetic nerve activity) in women with persistent functional abdominal pain. Karri et al [21] found that LF and HF decreased with no change in LF/HF in patients with chronic neuropathic pain induced by spinal cord injury, a finding that is similar to our study results. However, Hsu et al [22] found an increased InHF when the pain level decreased in the patients after total knee replacement, and they also found a decrease in InLF and LF/HF, which meant that the sympathetic nervous system was also involved in the pain. This was also demonstrated by the bioelectricity scores. We believe that both the sympathetic and parasympathetic nervous systems are involved in acute pain, while the parasympathetic nervous system plays an important role in chronic pain. In other words, sympathetic nerve activity can be adjusted during the duration of the pain. These results should be further investigated in future animal studies. Therefore, in addition to pain and function, silver needle therapy also improved the overall condition and ANS activity of patients with CNSLBP, especially the parasympathetic nervous system.

In this study, we chose physiotherapy as a control. Physiotherapy decreased the pain and improved the function in patients with CNSLBP after treatment. Physiotherapy also regulated autonomic nerve activity. However, the therapeutic effects of physiotherapy were not as effective as those of silver needle therapy, and we also found that 8 patients recovered pain at 1 month and 6 at 2 months. At 1 month, 50% of patients in the C group took medications to control the pain, while only 8% did so in the SN group. In total, physiotherapy was effective in 72% of patients (including patients taking medications), while silver needle therapy was effective in 100% of patients at the 6-month follow-up. Base on a review, Cuenca-Martínez et al [5] found that classical physiotherapy procedures were ineffective in the treatment of chronic nonspecific low back pain. Ghorbanpour et al. [23] reported that conventional physiotherapy was not as effective as exercise in reducing pain and improving function in patients with low back pain. The study showed that dry needling provided more therapeutic benefits compared with classical physiotherapy for patients with low back pain [24], although there was no follow-up period to evaluate the long-term effects. Therefore, for patients with CNSLBP, we speculated that physiotherapy may be significant in the short-term, but is associated with a certain degree of recurrence in the long term.

Silver needle therapy has many advantages. First, silver needle therapy is simple to operate and master, which is helpful in clinical applications. Second, silver needle therapy can be performed multiple times, although one part of the body can only be done once in 1–2 months because the tissues need 1–2 months to recuperate after the therapy. Finally, silver needle therapy decreased pain mainly through 3 mechanisms: elimination of sterile inflammation, improvement in blood circulation, and relief of muscle spasms, and it does not induce any side effects such as those caused by medications.

In this study, 5(10%) patients developed complications after silver needle therapy: 4 with ecchymosis and 1 with ambustion. As a minimally invasive procedure, for people with an abundant blood supply in the subcutaneous tissue, ecchymosis may develop that will be absorbed in a few days. This type of complication is difficult to avoid. There was also 1 patient with ambustion, which is related to the tilt of the needle’s body and its close proximity to the skin. We can prevent this complication by careful inspection, better fixation of the body of the needle, or placing gauze padding between the skin and the oblique needle.

Some limitations were noted in this study. First, the sample size was small, and the results need further support from investigating a larger sample. Second, in our country, medical insurance paid for all physiotherapy, while silver needle therapy was only partly paid for. Therefore, it was difficult to assess the possible effects of costs on the results in this study. Third, this study did not provide intervention mimicking the silver needle in the control group and the patients was not blinded, which may affect the results. Finally, the follow-up time was only 6 months, and a longer time might reveal more benefits or drawbacks.

## Conclusion

This study clearly provides a strong basis for the efficacy of silver needle therapy in patients with CNSLBP. Silver needle therapy alleviated pain, improved function, and regulated ANS activity for a long time without serious complications. Therefore, the use of silver needle therapy in the clinic should be promoted and it could be popularized in clinical practice.

## Data Availability

The data used to support the findings of this study are available from the corresponding author upon request.
